# Optimising pregnancy outcomes in patients with a history of eating disorders

**DOI:** 10.1186/2050-2974-2-S1-O11

**Published:** 2014-11-24

**Authors:** Leanne Barron, Warren Ward

**Affiliations:** 1 Brisbane City Doctors, Queensland University of Technology, Brisbane, Australia; 2 Royal Brisbane Hospital, Brisbane, Australia

## 

Infertility is a major long term complication of eating disorders, but even in cases where pregnancy is achieved, complications may ensue. Risks include early pregnancy loss, birth defects, eating disorder relapse and postnatal depression. This presentation will focus on recommendations for general practice management of pregnancy in patients with a history of an eating disorder.

Specific measures and investigations which may be useful for patients preparing for pregnancy include measurement of iron, zinc, vitamin B12, folate, vitamin D, calcium,magnesium and homocysteine levels. as well as measurement of thyroid function.

Supplements must be chosen carefully with regard to safety and potential risks of exacerbation of eating disorder symptoms. Dietary advice should include inclusion of full cream dairy products in order to optimise fertility and fetal development.

Medications may impact on fetal development, with sodium valproate lowering folic acid levels and SSRIs presenting an increased risk of pulmonary hypertension in the infant.

Nausea and vomiting in early pregnancy, as well as changing body shape, can trigger relapse symptoms, and psychological and nutritional support are required. Post partum depression and uncontrolled weight loss after pregnancy also present risks, requiring close monitoring and ongoing support.

This abstract was presented in the **Treatment in Community and Inpatient Settings** stream of the 2014 ANZAED Conference.

